# The difference in immune response and IL-12p35 methylation between newborns and adults

**DOI:** 10.1186/s12929-014-0076-0

**Published:** 2014-08-19

**Authors:** Chia-Jung Chen, Jia-Woei Hou, Bor-Luen Chiang

**Affiliations:** 1 Department of Pediatrics, Sijhih Cathay General Hospital, 2, Lane 59, Jiancheng Road, Sijhih City 221, Taipei, Taiwan; 2 Department of Pediatrics, Cathay General Hospital, 280 Section 4, Jen-Ai Road, Taipei 10630, Taiwan; 3 Graduate Institute of Immunology, National Taiwan University, No. 7 Chung-Shan South Road, Taipei 100, Taiwan; 4 Graduate Institute of Clinical Medicine, National Taiwan University, Taipei, Taiwan; 5 Allergy Center, National Taiwan University Hospital, Taipei, Taiwan

**Keywords:** Newborn, Interleukin-12, Methylation

## Abstract

**Background:**

The immune system of newborn is generally depressed by impaired production of Th1-cell associated cytokines, which results in increased susceptibility to intracellular pathogens and poor response to vaccinations. For avoiding abortion, the maternal and fetal immune systems tend to Th2-cell polarizing cytokines. Besides, IL-12p35 is a determining factor of the bioactivity of IL-12, which has an important role in the Th1 response. Recently methylated DNA is known to associate to inhibit transcription. Therefore, we explored the methylation status of CpG sites upstream of the coding sequence of the IL-12p35 gene to determine whether neonatal peripheral blood mononuclear cell (PBMC) synthesis lower level of IL-12 is related to methylated DNA.

**Results:**

PBMCs from adults expressed higher levels of IL-12p40 (p = 0.303) and IL-12p70 (p = 0.045) and had a strong ability to produce IL-12p35 mRNA (p = 0.01). However, there was no difference in the methylation status of CpG sites in the promoter of IL-12p35 between adults and newborns.

**Conclusions:**

We found that PBMC synthesis of bioactive IL-12p70 was significantly impaired in the neonatal period, potentially though a reduction in IL-12p35 production. The reeducation in IL-12p35 production might not be due to methylation of the promoter gene. But, the impairment of IL-12p35 expression during the neonatal period might be caused by other epigenetic regulation occurs in the chromatin level.

## Background

The immune system of newborns is functionally different from that of adults, and its immaturity results in an increased susceptibility to intracellular pathogens and poor response to vaccinations [1]. Inherent T cell defects contribute to the immature immunology of newborns, and these defects have been reported to be due to impaired tyrosine phosphorylation and hypermethylation of specific sites in the promoter region of the gamma interferon (IFN-γ) gene [2],[3]. Furthermore, many factors influence the Th1/Th2 imbalance in early life. The cytokines IL-12 and IL-4, acting through signal transducer and activator of transcription 4 (STAT4) and STAT6, respectively, are key determinants of the development into mature Th1 or Th2 cells.

IL-12 is a heterodimeric cytokine produced primarily by activated inflammatory cells (monocytes, macrophages, neutrophils, microglia and dendritic cells), and formed by a 35-kDa light chain (known as p35) and a 40-kDa heavy chain (known as p40). It is a pro-inflammatory cytokine that induces the production of IFN-γ, favors the differentiation of Th1 cells and forms a link between innate resistance and adaptive immunity. The p40 subunit of IL-12 is shared by IL-23, a cytokine with similar but also distinct actions compared with IL-12 [4]. IL-12p40 is produced in large excess over IL-12 heterodimer [5],[6]. Each gene encoding IL-12 is located on different chromosome. The highly coordinated expression of p40 and p35 to form the biologically active IL-12 (also called p70) in the same cell type at the same time is essential for the initiation of an effected immune response [7]. In the absence of IL-12p35, p40 is secreted as a monomer or a homodimer, whereas p35 can be secreted only when associated with p40 [7].

IL-12 has a central role in Th1 responses [5],[8]. Also, IL-12 has been shown to markedly suppress IL-4-induced IgE production by human mononuclear cells (PBMCs) *in vitro*[8]. These studies thus suggest that IL-12, through its capability of inducing Th1 cells, might be capable of down-regulating pathological Th2 responses. In addition, decreased level of IL-12 thus inhibits maternal Th1 activity which in turn protects the fetus during pregnancy. Therefore, IL-2, IL-12, and IFN-γ are very rarely detected in the feto-placental unit, and this will tend to bias the early life immune response towards a Th2 phenotype. Although evidences suggest that bioactive IL-12p35 mRNA is critical for the synthesis of bioactive IL-12p70, the regulation of IL-12p35 gene transcription remains poorly characterized [9]–[11]. Based on several studies, the nuclear factor kappa-light-chain-enhancer of activated B cells (NFκB) pathway activate by microbial antigens via Toll-like receptors (TLR 4 for lipopolysaccharide) in a MyD88-dependent manner [12],[13]. Then activated NFκB stimulates transcription of IL-12p35 and p40 gene, and the initial small amounts of IL-12 stimulate natural killer cells to produce IFN-γ [14],[15]. Together with activity of IFN-γ and lipopolysaccharide (LPS) also lead to the activation of Sp1 and its binding site in the p35 promoter as part of selective remodeling of a single nucleosome within the −310 to −160 region [16]. Together with innate and adaptive immune responses stimulate synergistically the production of IL-12 to sustain the inflammatory event and cell-mediated immunity against pathogens.

The immaturity of the mononuclear cell compartment contributes to impaired T cell responses in newborns [17],[18]. By analyzing dendritic cells (DCs) generated from adherent cord blood mononuclear cells cultured in the presence of GM-CSF and IL-4, the synthesis of the bioactive dimeric form of IL-12p70 has been shown to be profoundly impaired in newborn DCs [17]. The production of IL-12p40 in activated professional antigen-presenting cells is generally in great excess over that of the p35 chain, making p35 a limiting step in the formation of bioactive IL-12 [9],[10]. In addition, IL-12p35 gene expression is highly repressed in stimulated neonatal DCs, whereas the IL-12p40 gene expression was not altered. A recent study showed that the Sp1#1 binding site is located in a positioned nucleosome (nuc-2), which is selectively and rapidly remodeled upon activation of the p35 gene [16]. The molecular mechanisms responsible for deficient IL-12p35 gene expression in neonatal DCs is due to profoundly impaired nuc-2 remodeling in neonatal DCs in response to LPS [19].

There is also compelling evidence that DNA methylation has a repressive action on gene expression [20]. With regards to the immune system, the low capacity of naive adult T cells to produce IFN-γ has been associated with hypermethylation of CpG sites of the IFN-γ gene [3]. Therefore, the aim of this study was to explore neonatal PBMC immune responses by LPS stimulation *in vitro*, and to investigate the methylation status of CpG sites upstream of the coding sequence of the IL-12p35 gene to determine whether a similar phenomenon is involved in the deficient IL-12 synthesis by neonatal PBMCs.

## Methods

### Culture medium and reagents

The culture medium consisted of RPMI 1640 (Sigma-Aldrich, St. Louis M0, USA) supplemented with L-glutamine and 10% fetal bovine serum (Sigma-Aldrich). LPS from *Escherichia coli* (0128:B12) was purchased from Sigma-Aldrich. Recombinant human Il-12p40 and IL-12p70 were purchased from BD Biosciences (OptEIA ELISA sets, BD Biosciences, San Jose, CA).

### Subjects

Twenty-one normal full-term newborns delivered at the obstetric department of Cathay General Hospital, Taiwan, and 20 healthy laboratory volunteers over 20 years of age were recruited for this study. Heparinized peripheral blood was obtained from all of the newborns and adults. The exclusion criteria were: any systemic disorder that would affect immune status, current pregnancy or lactation, and the use of systemic antibiotics or anti-inflammatory medication within 6 months. This study was carried out with the approval of the Ethics Committee of Cathy General Hospital, and the parents or guardians of all of the children provided written informed consent.

### Cell preparation and culture

PBMCs were obtained from all samples by centrifugation over Ficoll-Hypaque gradients. The PBMCs were then cultured in the culture medium at 2 × 10^6^ cells/ml, and activated with LPS (100 ng/ml). After culture at 37°C under 5% CO_2_ for 24 hours, the culture supernatants were collected and stored at −20°C until analysis of cytokine production by enzyme-linked immunosorbent assay (ELISA). In the preliminary experiments, we determined that IL-12p70 and IL-12p40 levels reached a plateau at 24 hours after stimulation and did not increase further at 48 hours. For real-time PCR experiments, the cells were incubated for 3 hours and 6 hours.

### Cytokine determination

ELISA kits were purchased for the quantification of IL-12p40 and IL-12p70 (Pharmingen, BD OptEIA™). The detection limits were 10 pg/ml and 4 pg/ml, respectively. BD™ Cytometric Bead Array Th1/Th2/Th17 assays were conducted in the presence of a representative from the manufacturer. All kits included lyophilized standards that were reconstituted and diluted at 7 serial concentrations according to the manufacturer’s instructions. The standards included all recombinant cytokines tested, and were considered to be positive controls for the procedure. Bead fluorescence readings were done by flow cytometry (BD™ FACSCanto II).

### Quantification of IL-12p35 and IL-12p40 mRNA levels by real-time PCR

Total RNA was extracted using Trizol reagent (Invitrogen, Life Technology) and then reverse-transcribed into cDNA using random hexamers (SMART RT-PCR kit, BD Biosciences Clontech). Gene expressions were determined in triplicate by quantitative real-time PCR using SYBR Gene Expression Assays according to the manufacturer’s instructions on an ABI 7500Fast system (Applied Biosystems, Life Technology, CA, USA). Amplification of the endogenous control GAPDH was performed in order to standardize the amount of sample cDNA added. The oligonucleotide sequences used for IL-12p35 [GenBank: NM00082] and p40 [GenBank: NM002187] were: sense primers, 5’-CAAAACCTGCTG AGGGCCGTCA-3’ and 5’- ATGGAATTTGGTCCACTGATA-3’; antisense primers, 5’- GGAGGCCAGGCAACTCCCATTAG-3’ and 5’- TCAGGGTACTCCCAGCTG A-3’, respectively. Threshold cycle values (calculated by SDS Software for the ABI 7500 Fast system) were converted to the number of mRNA copies by comparison with the respective standard curve.

### DNA samples and bisulfite treatment

Samples of blood are collected in heparin-containing tubes, and the DNA was purified using a genomic DNA mini kit (QIAGENE, CA) following the manufacturer’s recommendations. The DNA was quantified using a NanoDrop ND-100 Spectrophotometer (Thermo Fisher Scientific, MA) prior to bisulfite treatment. The DNA (200 ng/reaction) was then treated with bisulfite using a QIAGENE® bisulfite kit (QIAGENE, CA), according to manufacturer’s instructions. The bisulfite-treated DNA was eluted twice in 10 μl of the manufacturer’s elution buffer (20 μl final volume) and stored in aliquots at −20°C until use.

### PCR and pyrosequencing

DNA methylation levels at the CpG sites were assessed using pyrosequencing (Pyromark Q24; Qiagen-Biotage). In brief, methylated cytosines were protected against a C → T transition following sodium bisulfite treatment (EpiTect Bisulfite Kit; Qiagen). Pyrosequencing is a quantitative sequencing method allowing for the quantification (%) of cytosine methylation levels at each CpG site of a given genomic region. The PCR primers were selected using the PyroMark Assay Design (version 2.0.1.15; Qiagen). The PCR and pyrosequencing primers for all three CpG islands tested within the IL-12p35 promoter gene locus were: IL-12p35①F: 5’- GGATATGTAAAGTGGGA GGTA-3’, IL-12p35①R: 5’- CCCACCACCCTAAATCCC-3’ (118 bp), and IL-12p35②F: 5’- GGGATTTAGGGTGGTGGG-3’, IL-12p35②R: 5’- TCCTTCT ATATCTCTCTCTACA-3’ (223 bp), and IL-12p35 Seq: 5’- CCCGGGAAAGTCCT GCCGCCGCGCCTCGGG-3’ (4 CpGs). Pyrosequencing was performed according to the manufacturer’s instruction. The PyroMark CpG Assays were used in conjunction with the PyroMark Q24 system (QIAGENE, CA), and each pyrosequencing reaction used 20 μl of PCR product. Pyrograms were analyzed in “Allele Quantification” mode in order to determine the percentage of C/T, corresponding to the percentage of methylated and unmethylated C at each of the CpG sites.

### Statistical analysis

The results were expressed as mean ± standard error of mean (SEM). Fisher’s exact test and the χ^2^ test were used to detect significant differences in categorical variables. Testing for differences in mean proportions was performed when appropriate with z tests. The non-parametric Wilcoxon’s rank sum test was used to detect significant differences in the median values of continuous variables. A two-tailed *P* value of 0.05 or less was considered to be statistically significant. The data were analyzed using the STATA SE 10.1 program (STATA Corp, College Station, TX, USA). Data for each kit were analyzed as recommended by the manufacturer. The cytokine concentration in each analyte was obtained by interpolating the fluorescence intensity of at least a 7-point dilution standard curve supplied with the kit and calculated by SoftMax® Pro Data Acquisition & Analysis Software and BD FCAP™ Array Software.

## Results

### Impaired IL-12 production by neonatal PBMCs

**Figure 1:**
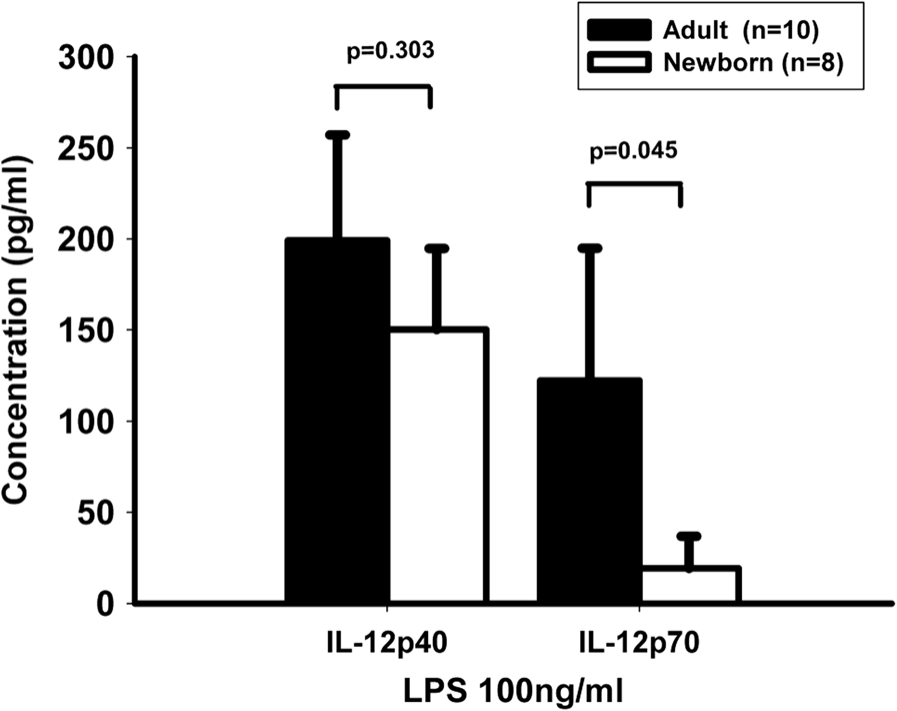
**Interleukin-12 production in peripheral blood mononuclear cells from adults and neonates.** Peripheral blood mononuclear cells from adults (n = 8) or neonates (n = 10) were cultured in a 48-well plate (2 × 10^6^ cells/ml) in the presence of 100 ng/ml of lipopolysaccharide. After 24 hours of stimulation, IL-12 production was evaluated in the culture supernatants using enzyme-linked immunosorbent assay. The results, obtained by subtracting the control values from the stimulated values, are expressed in pg/ml.

With LPS stimulation, the adult and newborn PBMCs produced comparable levels of IFN-γ, TNF and IL-6, whereas the synthesis of IL-17A was below detection levels in the newborn PBMCs and the production of IL-10 was higher in the adults (Table 1). With regards to IL-12, the levels of IL-12p40 secreted by the neonatal PBMCs were lower upon LPS activation (Figure 1). The most dramatic difference was observed for IL-12p70 which was produced at much lower levels by the neonatal PBMCs compared to the adult PBMCs under stimulation (p = 0.045). Therefore, we confirmed that the neonatal PBMCs were deficient in the synthesis of IL-12p70.

**Table 1:** Cytokine production by adult and neonatal PBMCs

**Cytokines (pg/mL)**	**IL-17A**	**IFN-γ**	**TNF**	**IL-10**	**IL-6**
**Adult**	2.58 ± 1.84	12.34 ± 7.34	124.38 ± 38.97	1416.74 ± 277.56	18461.11 ± 566.29
**Newborn**	0	1.37 ± 0.7	251.62 ± 47.18	304.28 ± 92.68	17074.1 ± 1116.47
**P value**	-	0.53	0.055	0.0017*	0.267

Data represent the mean with 2 standard deviations of at least 3 independent experiments on different donors. IL-17A, interleukin 17A; IFN-γ, interferon gamma; TNF, tumor necrosis factor; IL-10, interleukin 10; IL-6, interleukin 6. *P <0.05.

### Impaired IL-12p35 gene transcription in LPS-stimulated neonatal PBMCs

**Figure 2:**
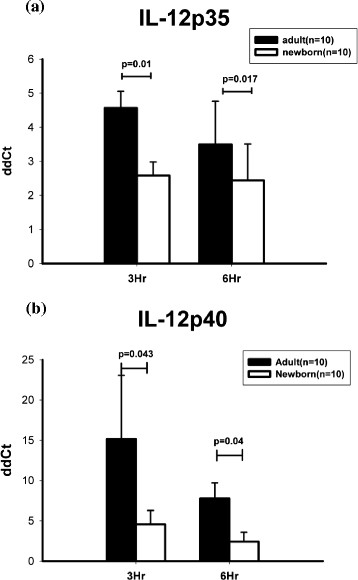
**Impaired IL-12p35 mRNA production in lipopolysaccharide (LPS)-stimulated neonatal peripheral blood mononuclear cells (PBMCs). (a)** IL-12p35 mRNA levels were determined by real-time PCR. The PBMCs were incubated with medium alone or stimulated with LPS (1 ng/ml) for 3 hours and 6 hours. IL-12p35 mRNA levels were normalized using endogenous control GAPDH and compared with the non-stimulated group. **(b)** The PBMCs were incubated with medium alone or stimulated with LPS (1 ng/ml) for the indicated period of time. IL-12p40 mRNA levels were assessed as described in **(a)**. The results are representative of at least 3 independent experiments performed with different donors.

The production of IL-12p40 and p35 mRNA in response to LPS stimulation alone was significantly lower in the neonatal PBMCs compared to the adult PBMCs, whereas IL-12p35 had a more significant effect (Figure 2). This indicated that deficient LPS-induced IL-12p70 synthesis in the neonatal PBMCs was related to impaired gene transcription of IL-12p35 in combination with IL-12p40.

### Difference in methylation patterns

**Figure 3:**
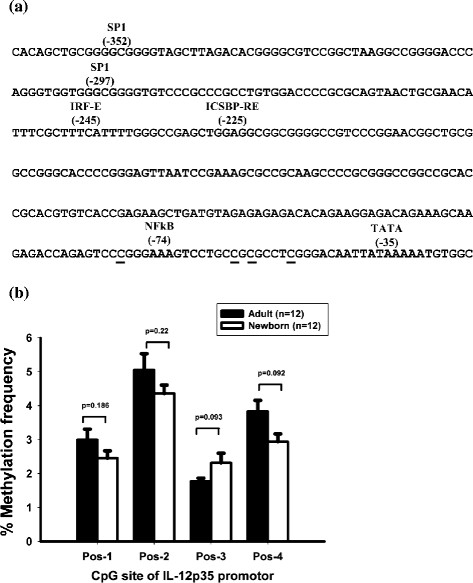
**Difference in methylation patterns. (a)** Sequence of human IL-12 p35 promoter. The sequences of the PCR used to perform pyrosequencing are indicated by underline. **(b)** Whole blood methylation % of IL-12p35 promoter region. Each graphic represents each CpG island (pos-1, 2, 3 and 4). The percentages of methylation are represented by bars. The black bars represent adult and the white bars represent newborn percentage of methylation of the IL-12p35 promoter site.

A total of 387 nucleotides, corresponding to the region −411 to −25 relative to the transcription promoter of the IL-12p35 gene were amplified. Application of the optimized conditions to generate IL-12p35 promoter methylation profiles for 34 CpG sites using whole blood DNA revealed that the methylation levels were extremely low. When focusing on the NFκB binding site, the pyrosequencing analysis indicated an absence of hypermethylation status on the IL-12p35 promoter region, with a methylation frequency of 2-6% along the promoter region (Figure 3).

## Discussion

The fetal and neonatal immune systems are strongly associated with physiological demands, which include protection against infection, avoidance of potentially harmful pro-inflammatory/Th1-cell-polarizing responses, and mediation of the transition between the normally sterile intra-uterine environment to the foreign antigen-rich environment of the outside world [21]. Mounting evidence indicates that the infection-induced production of pro-inflammatory/Th1-cell-polarizing cytokines, including interleukin-1β (IL-1β) and tumor-necrosis factor (TNF), is associated with premature labor and pre-term delivery [22]. The ability of pro-inflammatory cytokines to induce a spontaneous abortion is likely to be an important reason for the strong bias of the maternal and fetal immune systems of multiple mammalian species toward Th2-cell-polarizing cytokines [23],[24].

After birth, there is an age-dependent maturation of the immune response. During the prenatal and postnatal period, exposure to environmental microbial products can activate innate immunity which also accelerates the maturation process. This process occurs particularly if the exposure is repeated over a period of time [25], diminishing Th2-cell polarization and/or enhancing Th1-cell polarization and thereby reducing allergy and atopy, which is compatible with the hygiene hypothesis [26],[27]. Allergic disease such as atopic asthma has multifactorial origins, arising from complex interactions between genes and environment. Recent research has highlighted the possible influence of antenatal environment on the development of atopy and asthma [28]–[30]. The immunological processes are important during early infancy [31], when the immune system first directly exposed to environment antigens and establish the long-term responsiveness. Moreover, the degrees of functional competence of immunological system attained by birth, and the kinetics of the ensuing maturation process during infancy are important determinants of the nature of early immune response against antigens [32],[33] and of the risk of developing asthma and atopy later in life.

Previous studies have shown that the capacity of PBMCs to synthesize bioactive IL-12p70 is markedly impaired at birth and matures surprisingly slowly during childhood. The adult level of IL-12p70 synthesis is not acquired until the age of twelve [34]. Indeed, using real-time PCR technique, we showed IL-12p35 gene expression was depressed in neonatal mononuclear cells to that of adult. It has become clear that IL-12 is not only required during the initial phases of Th1-cell polarization, but that it is also required to maintain the efficiency of the IFN-γ transcription machinery in Th1 cells [35]. Moreover, the production of IL-12 heterodimer by DCs seems to be much less dependent on the presence of IFN-γ and of signals from T cells than is production by phagophages, although production of IL-12 heterodimer is facilitated by stimulation through CD40L [11]. Neonatal T cells proliferate relatively poor when stimulated with isolated allogeneic neonatal DCs but strongly with adult DCs [36]. While the intrinsic properties of T cells undoubtedly contribute to immunological immaturity during the neonatal period [2],[37], it is also clear that many of the “deficiencies” observed in neonatal immune function are dependent on antigen-presenting cells (APCs) [38]. Similarly, the reduced capacity of neonatal T cells to produce Th1-cell-polarizing cytokines, IFN-γ, is markedly improved when cultured with adult, rather than cord APCs [39]. These results suggest that neonatal DCs and APCs lack the capacity to deliver important Th1-cell-polarizing signals to T cells.

The reasons of these insufficiencies might due to the both quantitative and qualitative differences between DCs, monocytes and APCs from newborn and adults. Qualitative differences in monocytes are evident in utero, as third-trimester phenotyping of fetal or neonatal circulating monocytes by flow cytometry has revealed that human fetal monocytes express lower levels of major histocompatibility complex class II molecules, which potentially contributes to impaired APC activity [40]. According to above statement, the reduced inflammatory cell and Th1-cell-polarizing fetal APCs activity are thought to be necessary to reduce the risk of alloimmune reactions between mother and fetus, and phenotypic and stimulus-specific functional immaturities have been reported to be present at birth in both mice and humans [41],[42].

Eukaryotic chromatin structure modification of some cytokines genes can occur during development and then persist in the differentiated cell. In human monocytes, priming of IL-12p35 synthesis was shown to rely on an alternative TATA-dependent promoter. A constitutively active CpG-rich promoter region was identified 5’ of the TATA box in EBV-transformed lymphoblastoid cells [43]. Although, the difference of CpG hypermethylation was not found between newborns and adults in this report, impaired nucleosome remodeling of newborn was found preceded IL-12p35 mRNA synthesis in previous report. [16] Many aspects of transcription-factor binding to relevant cis-acting elements of IL-12p35 promoter are similar in the DCs from adults and newborns, but chromatin-accessibility assays revealed that the LPS-induced nucleosome remodeling, required for the effective functioning of the upstream Sp1 transcription factor sites, was substantially impaired in the neonatal DCs. Therefore, neonatal IL-12p35 gene transcription is repressed at the chromatin level. Of note, the administration of IFN-γ has been reported to be able to restore both nucleosome remodeling and IL-12p35 gene transcription in vitro, indicating that modulation of nucleosome remodeling is essential in the activation of neonatal DCs [19]. Several researches at the murine IL-12p35 locus revealed that transcription of IL-12p35 is regulated by chromatin, as a positioned nucleosome at the promoter overlaps the transcription factor biding sites [44],[45]. Kobayashi *et al.* used wild type and IL-10 ^−/−^ mice model to investigate the mechanisms IL-10 inhibits IL-12p35 expression [45]. The results revealed that histone deacetylation on the IL-12p35 promoter might mediate homeostatic effects of IL-10 in macrophages. These results seems that histone deacetylases has an important role of regulation IL-12p35 nucleosome modification at the promoter site. Further investigation about IL-12 nucleosome modification of newborn could focus on histone deacetylation.

Otherwise, our result showed neonatal PBMC produced higher IL-10 by LPS stimulation. Several studies have demonstrated higher IL-10 responses in cord blood compared to adult samples [46],[47]; however other studies have revealed controversial results [48],[49]. Vosters et al. demonstrated that the IL-10 response elicited by LPS was reduced significantly in cord blood mononuclear cells compared to adult PBMCs [49], and the deficient production of IL-10 in response to LPS persisted until 18 months of age. In addition, Belderbos et al. indicated that adult PBMCs produced higher levels of IL-10 in response to LPS when stimulated in the presence of neonatal plasma than in the presence of adult plasma [47]. Therefore, the higher IL-10 responses in cord blood may also reflect the impact of factors present in the plasma [49],[50]. In this study, we also showed that isolated PBMCs which stimulated by LPS produced IFN-γ, TNF and IL-6 with no significant difference between newborns and adults.

## Conclusions

Childhood clearly represents a critical period in the development of immune responsiveness, with important implications for the development of both infectious and allergic disorders [34]. In order to gain insight into the mechanisms responsible for the impaired Th1 responses in childhood, we examined the methylation status of IL-12p35 at the promoter region. The results showed that the capacity of PBMCs to synthesize bioactive IL-12p70 was markedly impaired in the post-natal period, and that this was contributed to by depressed IL-12p35 mRNA production. This pattern of reduced IL-12p35 production was observed in response to stimulation with LPS that initiated the IL-12 gene expression via distinct mechanisms. The mechanism of development of IL-12-producing capacity throughout childhood remains unclear, however methylation of the promoter region did not contribute to this suppressed function of IL-12p35.

This type of defect may result from other epigenetic rather than methylation mechanisms, and may take place in the chromatin level. Both DNA methylation and histone modifications work together to activate and silence gene by influencing chromatin structure, and also determine when and where a gene is expressed during development. Exploration the influence of histone deacetylases on IL-12 gene expression will be the next step. With further research and understanding of this defect and the continued development of pharmacological agents that modify chromatin, epigenetics may become a suitable therapeutic approach for the treatment of inflammatory cell pathology in asthmatic and allergic patients.

## Competing interests

None of the authors have professional, personal, or financial competing interest to report.

## Authors’ contributions

CJC collected information, designed and organized the structure of the contents and wrote the manuscript. JWH, BLC reviewed literature, discussed and suggested the contents as well as edited the manuscript. All the authors read and approved the final manuscript.
